# Interacting stressors matter: diet quality and virus infection in honeybee health

**DOI:** 10.1098/rsos.181803

**Published:** 2019-02-06

**Authors:** Adam G. Dolezal, Jimena Carrillo-Tripp, Timothy M. Judd, W. Allen Miller, Bryony C. Bonning, Amy L. Toth

**Affiliations:** 1 Department of Entomology, University of Illinois, Urbana-Champaign, IL 61820, USA; 2 Department of Ecology, Evolution, and Organismal Biology, Iowa State University, Ames, IA 50011, USA; 3 Department of Plant Pathology and Microbiology, Iowa State University, Ames, IA 50011, USA; 4 Department of Entomology, Iowa State University, Ames, IA 50011, USA; 5 Department of Biology, Southeast Missouri State University, Cape Girardeau, MO 63701, USA

**Keywords:** honeybee, nutrition, Israeli acute paralysis virus, micronutrients

## Abstract

Honeybee population declines have been linked to multiple stressors, including reduced diet diversity and increased exposure to understudied viral pathogens. Despite interest in these factors, few experimental studies have explored the interaction between diet diversity and viral infection in honeybees. Here, we used a mixture of laboratory cage and small semi-field nucleus hive experiments to determine how these factors interact. In laboratory experiments, we found that high-quality diets (polyfloral pollen and high-quality single-source pollen) have the potential to reduce mortality in the face of infection with Israeli acute paralysis virus (IAPV). There was a significant interaction between diet and virus infection on mortality, even in the presence of high virus titres, suggesting that good diets can help bees tolerate virus infection. Further, we found that extreme stress in the form of pollen starvation in conjunction with IAPV infection increase exiting behaviour from small experimental hives. Finally, we showed that higher-quality pollen diets have significantly higher iron and calcium content, suggesting micronutrient deficiencies could be an under-explored area of bee nutrition.

## 1. Background

The health of managed honeybees has been of substantial concern, with yearly losses annually remaining higher than beekeepers consider sustainable [1,2]. The increased costs associated with beekeeping put strain on the industry and could affect the downstream costs of production for bee-dependent crops and ecosystem services [3,4]. Honeybee declines have been linked to a number of factors, but the current consensus is that widespread losses are influenced by variable but interacting environmental stressors, specifically pesticide exposure, pressure of pests and pathogens, and poor nutritional resource availability [5–7].

Despite the importance of these interactions, there are still few studies to date that experimentally test how honeybees respond to concurrent stressors. In many areas around the globe, there have been extreme changes in land usage associated with urbanization and industrialized agriculture [8]. From a pollinator's perspective, this transformation of the land has generally resulted in lower diversity floral resources [9]. Lower flower diversity creates challenges in finding enough food, particularly a well-balanced and diverse diet for generalists. Honeybees are generalists, and previous research has shown that honeybees thrive on a diet that includes a large diversity of floral resources and prefer mixed pollen when given a choice between polyfloral and monofloral pollen [10,11].

Pollen nutritional content is highly variable. The pollen of some species of flowers lacks key nutrients [12] necessary for honeybee nutritional needs [13]. This includes the pollen of numerous crops [14] that depend heavily on honeybees for their pollination. The use of honeybees for concentrated pollination of a single crop can result in pollen diets that lack key nutrients, leaving colonies weakened and in need of nutritional supplementation [14]. Recent studies have also found connections between landscape structure and honeybee health. Areas with more developed land (e.g. due to urbanization and monoculture crop production) were associated with greater colony loss compared to areas with more open, undeveloped land [5]; similarly, uncultivated land has been positively associated with honey production and survival [15] and physiological health [16–18]. Further, as agricultural intensification continues, once profitable areas have become decreasingly suitable for supporting apiaries [19,20]. Landscape management strategies that aim for a diverse range of floral sources near managed hives have the potential to offset some of these problems [17].

Studies in a wide variety of insects have shown that nutritional stress can lead to changes in susceptibility to infection by bacteria and viruses, impacting the severity of symptoms of these pathogens [21–23]. In bees, there are documented relationships between poor nutritional status, reduced survival, reduced immunity and increased susceptibility to pathogens and parasites. For example, caged bees fed on monofloral pollen had reduced lifespans and higher mortality than bees fed on polyfloral pollen sources [10,11,14]. Poorly nourished bees are more susceptible to *Nosema ceranae*, a microsporidian gut pathogen; specifically, bees fed a polyfloral blend or a high-quality monofloral pollen have lower *N. ceranae* levels and longer lifespans than bees fed lower quality monofloral diets [24]. Bees fed on monofloral food sources have reduced glucose oxidase activity, potentially indicating reduced immune response [25]. Further, bees fed protein-deficient diets have been found with higher titres of deformed wing virus (DWV) [26].

While protein, fat, amino acids, and even phytochemicals [27] have received thorough attention, there has not been as much research on the role of micronutrients, like calcium, magnesium, sodium, etc., in honeybee diets [28]. Some previous studies have identified their importance, particularly in brood rearing [29,30], but there has not been exploration of how these nutritive components could contribute to pathogen resistance or susceptibility in bees [12]. The importance of micronutrients in maintaining immunity is well studied in mammals [31], and the importance of these elements in cellular processes and immunity has been observed in some model insect systems [32–34]. Therefore, a better understanding of how micronutrients contribute to honeybee immunity can help identify how different dietary components affect different aspects of bee health [28].

Bee nutrition is only one of the many environmental stressors that impact honeybee colonies. Honeybee viruses, of which more than 20 are known [35], are widespread and often persist as asymptomatic infections, even in otherwise healthy colonies [36]. Bee viruses were a relatively minor problem until the spread of the parasitic mite, *Varroa destructor* [37], which supports replication of some viruses and serves as a virus vector [38–40], delivering high viral titres to mite-infested bees with severe pathological consequences [41]. Israeli acute paralysis virus (IAPV) causes infected bees to exhibit shivering wings followed by paralysis and death [42]. Infection with IAPV can cause high acute mortality [42,43], and IAPV has been associated with large-scale colony losses, like colony collapse disorder (CCD) [44].

Despite the increasing interest in honeybee viruses and the effects of nutritional environment, there has been little experimental work to understand how these factors interact [45]. We hypothesized that honeybees are better able to resist the pathogenic effects of virus infection when provided with a higher-quality, polyfloral pollen diet. To test this, we used experimental infection of honeybees with a well-characterized virus inoculum [43], combined with controlled pollen diets, to test how different pollen diets affect response to IAPV infection. To begin testing the effects of these interactions in more natural settings, we also measured the proportion of bees that left the colony following each treatment. Studies of how environmental stressors interact to affect bee health have revealed that such stressors can be synergistic [46], and thus identifying and targeting interacting stressors may be one of the best ways to improve bee health. The goal of this work was to use an integrative approach using field, laboratory and physiological methods to reveal some of these interactions and open up this topic for further study.

## 2. Material and methods

### 2.1. Virus inoculum production

Honeybees were treated with a virus inoculum identical to that thoroughly described in Carrillo-Tripp *et al*. [43] and electronic supplementary material. Because no virus-free cell culture existed, at the time of these experiments, and honeybee pupae always have some background virus levels, it was not possible to produce pure IAPV particles. To calculate the percentage of each virus in virion preparations (acute bee paralysis virus (ABPV), black queen cell virus (BQCV), DWV, IAPV, Kashmir bee virus (KBV) and sacbrood virus (SBV)), the virus stock was diluted 1 : 1000 and used directly for RTq-PCR (as described below). This inoculum contained 7.7 × 10^6^ genome equivalents (g.e.) (97.94%) SBV, 7.84 × 10^4^ (0.98%) IAPV, 6.16 × 10^4^ (0.77%) DWV, and 2.57 × 10^4^ (0.32%) BQCV. ABPV was below detectable limits. Despite the high presence of other viruses, particularly SBV, this inoculum results in high acute mortality and an increased titre of only IAPV [43].

### 2.2. Infection and feeding of caged honeybees

In July 2014, one or two frames of emerging brood were removed from 15 healthy colonies housed in the Iowa State University research apiary. The following day, newly emerged bees from all frames were shaken into a large tub and gently mixed to provide a homogenized mixture of bees from the 15 different colonies. Bees from this mixture were then counted out into clear acrylic cages (10.16 × 10.16 × 7.62 cm) in groups of 35 per cage. Cages were randomly assigned to treatments and kept in a single walk-in insect rearing room kept at 32–34°C and 50% relative humidity. Each cage received a virus treatment (control 30% sugar solution or the same sugar solution with virus inoculum added) and a pollen dietary treatment (no pollen, polyfloral pollen, *Cistus* sp. (rockrose) pollen, or *Castanea* sp. (chestnut) pollen).

For the virus treatment, cages were provided an open feeder containing either 0.6 ml 30% sucrose solution or the same solution containing a 1 : 1000 dilution of viral inoculum (described in detail in Carrillo-Tripp *et al*. [43]). This dose was chosen through the production of dose–response curves to identify an inoculum concentration that would result in intermediate levels of mortality, as described in Carrillo-Tripp *et al.* [43]. Bees had ad libitum access to these feeders for 12 h, after which all of the solution had been completely consumed, and the open feeders were removed. For the rest of the experiment, bees had ad libitum access to untreated 30% sucrose solution fed through a drip feeder at the top of the cage.

Concurrent with the introduction of the open feeder, each cage received a pollen treatment. The cage either had no pollen added, polyfloral pollen, *Cistus* pollen, or *Castanea* pollen introduced into the bottom of the cage. *Cistus* and *Castanea* pollen were purchased from Pollenergie^®^ (France). These pollens have been nutritionally well characterized, with *Cistus* being of overall lower quality (lower protein, amino acid content) and *Castanea* being of high quality (higher protein, amino acid content, beneficial affects during *Nosema* challenge) [24]. The polyfloral blend, identical to that described in Dolezal *et al*. [47], contained more than five species of pollen, with the most abundant being dandelion (*Taraxacum* sp. L.) and willow (*Salix* sp. L.), each of which made up approximately 8% by mass of the total blend. To this mixture, *Cistus* and *Castanea* pollen were added to a total of 8% by mass for each. In all cases, pollen was bee-collected and received in corbicular pellets. Each pollen source was homogenized into a powder in a coffee grinder, weighed out into 0.2 g aliquots and added to the bottom of each cage. After 24 h, remaining pollen was removed and replaced with fresh. In all cases, bees did not consume all of the pollen in any given 24 h period.

Mortality was monitored in all cages every 12 h with dead bees removed at each interval. Previous experiments had shown that mortality occurs primarily between 36 and 48 h post-infection (hpi, [43]) with some cages devoid of live bees by 72 hpi. Therefore, to sample bees during the height of infection but before death, six live bees were removed from each cage at 36 hpi. Mortality effects were measured as a cumulative percentage at 72 hpi, after which the experiment was ended because some cages had too few bees for meaningful analysis, similar to previous results [43]. Collected samples were stored at −70°C until processing.

### 2.3. Infection and feeding of nucleus colonies

In August 2014, eight single cohort colonies were produced from bees derived from 14 different colonies. Newly emerged workers from each colony were mixed together to create a single randomized pool of bees. From this pool, 1200 bees were counted, marked on the thorax with paint (denoting treatment for identification), and introduced to a five-frame nucleus hive body. Each queenless nucleus colony (i.e. a small colony containing the ‘nucleus’ of a larger colony) received bees marked with the same colour, allowing bees to be identified by treatment within and between hives. Each hive body contained two frames of drawn comb into which 20 ml of 30% sucrose solution had been added to the centre cells. Below those cells, 7 g of pollen were added directly into other cells. This approach allowed the bees to walk and feed directly from the comb, more similarly to a normal colony, but with a controlled quantity of food. Two colonies each were fed *Castanea* pollen, *Cistus* pollen, polyfloral pollen, or no pollen, giving a total of eight nucleus colonies; all received the same 30% sucrose solution diet. Pollen treatment was from identical source and processing as described for the cage experiments.

Colonies were then placed into an enclosed, bisected mesh greenhouse, with one colony of each pollen treatment on each side; because of the pollen delivery method, there was not any transference of pollen between colonies. Bees from each side could not interact with each other, preventing spillover of virus between infected and uninfected hives. The order of the colonies in the greenhouse was chosen at random, but was identical for different sides of the greenhouse. For the first seven days, colony entrances were sealed with a screen to prevent movement of bees and control diet intake. Every two days, extra sucrose solution and pollen diet (if applicable) was replenished or added to provide for ad libitum feeding and colony population estimated. In no cases was the food source exhausted in this interval. Population estimates where achieved by photographing each frame and then counting the number of bees in each image. On day 7, the feeding frames were removed from each colony. Then, one colony from each pollen treatment, all on the same side of the bisected greenhouse, received 20 ml of 30% sucrose solution containing the 1 : 1000 virus inoculum at identical concentration as used in the cage experiments. Further, 20 ml represented an almost identical scale-up of the volume used in the cage experiments. The other colony from each pollen treatment, on the other side of the greenhouse, received control sucrose solution. At this point, pollen feeding also ceased, as honeybee workers only feed on pollen for the first six to eight days post-emergence. On day 8, colony entrances were opened to allow honeybees to orient and forage. Feeder stations of 30% sucrose solution were placed 4 m from the hive, in the centre of the greenhouse.

To observe a metric for foraging behaviour in each colony, each hive was observed mid-morning and mid-afternoon each day for seven days. Each observation period consisted of two 15 min intervals per hive, cycling between the two sides of the greenhouse, with the starting side rotated each day. Hive-exiting behaviour was estimated by counting the bees observed leaving the colony entrance and flying towards the feeder station. Arriving behaviour was concurrently recorded, though this value was confounded by bees performing orientation behaviour (i.e. hovering in front of the entrance). Every other day, the colonies were quickly opened and the combs photographed to allow counting of bee population. To estimate hive-exiting and arriving, the observed exiting or arriving bees was divided by the population on the nearest recorded day, resulting in proportion of each colony. Days with rain, which prevented any bees from leaving, were omitted from analyses. This experiment was repeated three independent times, for total of 24 nucleus colonies, three per each pollen diet/virus treatment.

### 2.4. Virus quantification

From the cage experiments, two bees were sampled at 36 hpi, pooled, and processed from 9 to 10 randomly chosen cages (four or five from each replicate). Virus titres were measured via RT-qPCR identically to the methods described in [43] and in the electronic supplementary material.

### 2.5. Lipid quantification

To quantify total lipids, a metric for colony health associated with foraging behaviour and immunity [48], four bees collected from each cage collected at 36 hpi had their gut content removed, and were then homogenized in liquid nitrogen into a powder. Approximately 100 mg subsamples from this homogenate were precisely weighed and then processed for lipid quantification using a phosphor-vanillin spectrophotometric assay optimized for bees [48]; lipid per gram was then calculated based on the mass of the subsample.

### 2.6. Micronutrient quantification

From the solid homogenate of bee tissue produced for lipid quantification, from the cage experiments, another approximately 100 mg subsample was collected and analysed for concentration per sample mass of Ca, Cu, Fe, K, Mg, Mn, Na and Zn as described in [49] and electronic supplementary material.

### 2.7. Statistical analysis

To test for interaction between diet and virus treatment on mortality in cages, comparisons were made using a mixed model ANOVA by the ‘lme’ function from the R package ‘nlme’ with virus treatment and diet treated as separate fixed factors and cage as a random factor. After a significant interaction was observed, a separate mixed model ANOVA was performed using each iteration of virus + diet as a treatment by pairwise planned Fisher LSD comparisons within the virus treatment groups (i.e. between infected groups and uninfected groups). We also contrasted virus-treated groups with their same-diet uninfected counterparts to verify infection occurred. These comparisons were then corrected with the Benjamini–Hochberg false discovery rate procedure [50] to limit familywise Type I error rates [51]. An identical procedure was used to analyse differences in virus titre, with the exception that a log transformation was performed on these data to allow them to fit a normal distribution. To identify how each virus + diet treatment affected lipid content and micronutrient concentrations, an identical mixed model approach was performed; then, a separate mixed model ANOVA was performed using each iteration of virus + diet as a treatment followed by a Tukey's HSD test. For nucleus hives, there was no way to statistically compare changes in mortality with a low hive-level sample size, but per cent change in population is numerically reported. Foraging activity, as estimated by the proportion of bees leaving a colony during each observation period, was compared using a repeated-measures ANOVA with colony as a random effect, using the ‘lme’ function in R followed by a Tukey's HSD post-hoc comparison.

## 3. Results

### 3.1. Diet can partially prevent mortality due to virus infection

**Figure 1.**
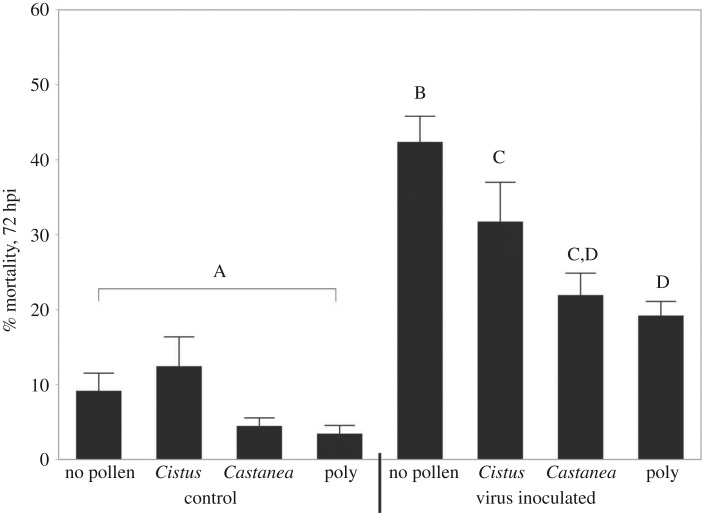
Per cent mortality in cages fed different diets (no pollen, *Cistus*, *Castanea*, polyfloral mixture) and treated with either control sucrose solution or a viral inoculum at 72 hpi. Mean ± s.e., letters denote significant differences between contrasted groups; mixed model ANOVA followed by Fisher LSD, Benjamini–Hochberg correction, *p* < 0.05.

Bee mortality at 72 h post-inoculation (hpi) differed among the treatment groups (mixed model ANOVA across all treatment groups, d.f. = 7, 108; *F* = 19.28; *p* < 0.0001). There was a significant interaction of virus and diet treatment (mixed model ANOVA, d.f. = 3, 108; *F* = 3.311, *p* = 0.023), with individual significant effects of both virus (mixed model ANOVA, d.f. = 1, 108; *F* = 95.26; *p* < 0.0001) and diet treatment (mixed model ANOVA, d.f. = 3,108; *F* = 9.93; *p* < 0.001). In all cases, virus-treated cages showed significantly higher mortality uninfected diet counterparts (Fisher LSD, Benjamini–Hochberg correction, *p* < 0.05). Without virus exposure, none of the treatment groups showed differences in mortality (*p* > 0.05). Within the virus-treated groups, bees fed no pollen showed higher mortality than all other groups (Fisher LSD, Benjamini–Hochberg correction, *p* < 0.05). Between the pollen-fed groups, bees fed polyfloral pollen exhibited significantly lower mortality than bees fed only the *Cistus* pollen (*p* = 0.03); *Castanea*-fed bees exhibited intermediate mortality, which was not significantly different than either polyfloral (*p* = 0.62) or *Cistus*-fed bees (*p* = 0.083; figure 1).

### 3.2. Diet does not affect IAPV levels in caged bees

**Figure 2.**
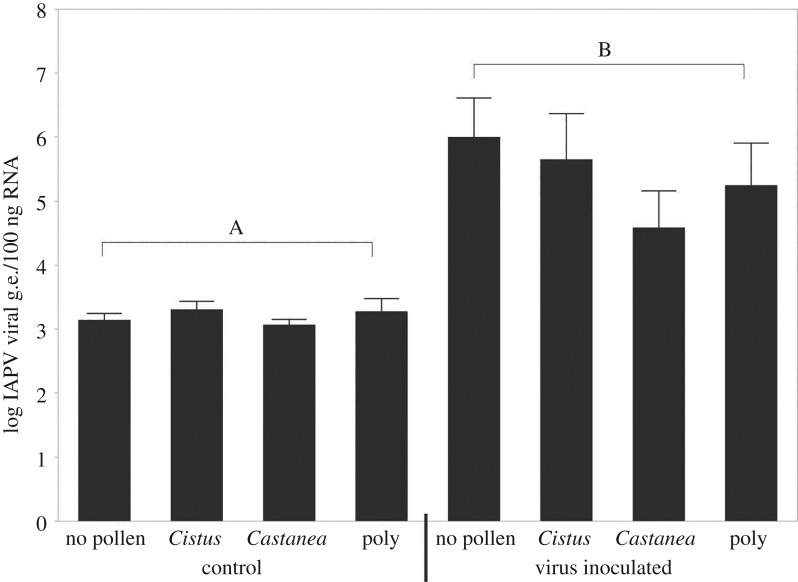
Virus load of IAPV, expressed as mean ± s.e. log viral genome equivalents (g.e.) in 100 ng of total RNA at 36 hpi from a pool of two live bees sampled per cage. Mean ± s.e., letters denote significant differences between groups (mixed model ANOVA, Fisher LSD, Benjamini–Hochberg correction, *p* < 0.05).

A previous study using the same virus inoculum found that treatment resulted in elevation of both IAPV and SBV titres compared to controls, but that only IAPV titres increased over time, showing that IAPV is the main cause of mortality [43]. Here, we obtained similar results, with virus treatment not affecting BQCV (ANOVA, d.f. = 1,7; *F* = 1.53, *p* = 0.17, electronic supplementary material, figure S1a) or DWV (ANOVA, d.f. = 7, 68; *F* = 0.32; *p* = 0.941, electronic supplementary material, figure S1b) levels. However, IAPV (ANOVA, d.f. = 1,7; *F* = 6.58, *p* < 0.0001, figure 2) and SBV (ANOVA, d.f. = 1,7; *F* = 9.09, *p* < 0.0001) were significantly different between groups (figure 2; electronic supplementary material, figure S1c), with inoculation significantly raising IAPV and SBV titres over background (ANOVA, d.f. = 1,74, *p* < 0.0001; Fisher LSD, Benjamini–Hochberg correction, *p* < 0.05). Although both IAPV and SBV levels were elevated after the infection treatment (electronic supplementary material), a previous study using the same methodology and examining virus dynamics over time suggests the mortality effect is primarily due to IAPV [43]. Within virus treatment groups, there were also no significant differences in IAPV or SBV titres (Fisher LSD, Benjamini–Hochberg correction *p* > 0.05; figure 2, electronic supplementary material, figure S1c).

### 3.3. Diet affects virus-induced mortality

**Figure 3.**
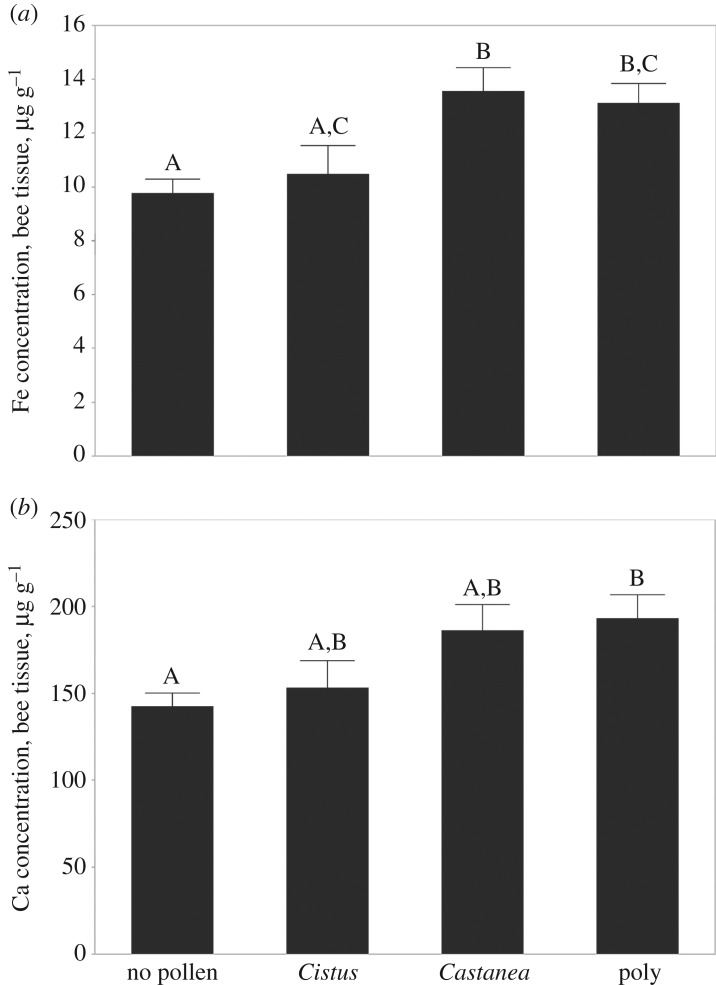
Micronutrient concentration of (*a*) iron (Fe) and (*b*) calcium (Ca) in µg g^−1^ bee mass. Mean ± s.e., letters denote significant differences between groups (mixed model ANOVA, Tukey's HSD, *p* < 0.05).

There were no differences in bee tissue levels of Cu, K, Mg, Mn, Na or Zn among the pollen diets (ANOVA, *p* > 0.05). There were differences, however, in Fe (ANOVA, d.f. = 3,70; *F* = 5.66, *p* = 0.0016, figure 3*a*) and Ca (ANOVA, d.f. = 3,70; *F* = 4.03; *p* = 0.0105, figure 3*b*) content (µg nutrient/g bee mass). Polyfloral-fed bees (Tukey's HSD, *p* = 0.019) and *Castanea*-fed bees (Tukey's HSD, *p* = 0.0028) had significantly higher Fe content than bees fed no pollen. *Castanea*-fed bees also exhibited higher Fe content than *Cistus*-fed bees (Tukey's HSD, *p* = 0.029; figure 3*a*). Polyfloral-fed bees showed a marginally higher, though not significantly different, iron concentration than *Cistus*-fed bees (Tukey's HSD, *p* = 0.09; figure 3*a*).

Ca content was significantly higher in polyfloral-fed bees than those fed no pollen (Tukey's HSD, *p* = 0.015), and *Castanea* pollen-fed bees showed marginally higher Ca content than bees fed no pollen (Tukey's HSD, *p* = 0.073), as did polyfloral compared to *Cistus*-fed bees (Tukey's HSD, *p* = 0.088; figure 3*b*). Micronutrient content was not, however, predictive of either mortality or virus titres (mixed model ANOVA, *p* > 0.05). There was no difference in lipid content between bees of any groups (ANOVA, d.f. = 7, 71, *F* = 1.48; *p* = 0.18).

These results mirror the patterns found in the pollen types. Ca concentration was 588 µg g^−1^ in polyfloral pollen, 658 µg g^−1^ in *Castanea* pollen, and 149 µg g^−1^ in *Cistus* pollen; that is, *Cistus* contained less than 25% of the Ca found in the other pollens. Fe concentration was 23.3 µg g^−1^ for polyfloral pollen, 17.9 µg g^−1^ for *Castanea* pollen, and was undetectable for *Cistus*.

### 3.4. Colonies under maximum dietary and infection stress exhibit elevated hive-exiting behaviour

**Figure 4.**
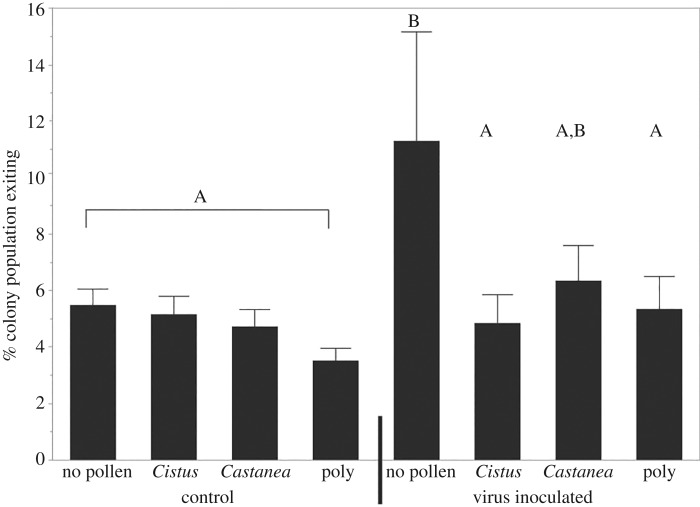
Per cent of a colony's population observed exiting the hive entrance during 30 min observation periods repeated over 6 days per colony. Mean ± s.e., letters denote significant differences between groups (repeated-measures ANOVA, Tukey's HSD, *p* < 0.05).

Behavioural observations revealed significant differences by treatment in the proportion of a colony exiting the hive (repeated-measures ANOVA, d.f. = 7, 42; *F* = 2.87; *p* = 0.0154, figure 4) and arriving to the hive (repeated-measures ANOVA, d.f. = 7,42; *F* = 2.80; *p* = 0.0172, electronic supplementary material, figure S2), which together form a rough metric for quantity of the colony dedicated to foraging. The differences in hive-exiting activity were driven by no pollen + virus-treated group, which exhibited significantly more bees exiting the hive than all other groups (Tukey's HSD, *p* ≤ 0.05; figure 4). The no pollen + virus group showed significantly more arriving behaviour than the uninfected polyfloral group (Tukey's HSD; *p* < 0.05) and marginally (but not significantly) more arriving behaviour than the groups receiving *Castanea* + no virus (Tukey's HSD; *p* = 0.075) and *Castanea* + virus (Tukey's HSD; *p* = 0.068; electronic supplementary material, figure S2).

Populations of all colonies decreased over the course of the experiment (15 days), and the proportion decrease provides a rough estimate of mortality. The mean per cent population decreases were no pollen + no virus, 64%; no pollen + virus, 78%; *Castanea* + no virus, 40%; *Castanea* + virus, 44%; *Cistus* + no virus, 30%; *Cistus* + virus, 45%; polyfloral + no virus, 37%; polyfloral + virus, 42%. With the sample sizes available, we lack adequate power to statistically compare population decrease data between colony treatment groups. Numerically, colonies fed no pollen or *Cistus* pollen showed greater population decreases when also treated with virus. Bees fed no pollen also exhibited the highest overall population decreases of any of the groups, even in the absence of virus inoculum.

## 4. Discussion

In this study, we observed that interactions between two important environmental stressors, diet and viral infection, can have a substantial effect on bee mortality and also have the potential to disrupt colony division of labour. We demonstrated that it is not simply food limitation, but food quality, that can be a serious factor in bees' resilience to other forms of stress. Our data support the hypothesis that pollen diet quality can affect honeybee response to viral infection. While other studies have shown that immunity [25,52,53] and microsporidian parasitism [24] are affected by diet, this is the first evaluation of the effects of differential dietary inputs on mortality, virus level and behaviour in experimentally virus-infected honeybees.

Our results show that diet quality, not just quantity, is a driving factor in bee health. We showed that pollen sources have the potential to differentially affect bees’ ability to tolerate viral infection, even when bees had ad libitum access to each diet. Di Pasquale *et al*. [24] showed similar effects with Nosema parasitization, but did not allow ad libitum feeding (to prevent bees from compensating for low-quality diets by consuming more). Therefore, their results did not rule out that effects could have been exacerbated by a restricted amount of pollen.

Both the presence of pollen in the diet and the nutritional composition of pollen matter for resilience against virus infection. Bees fed no pollen exhibited the highest virus-induced mortality, unsurprisingly showing that full starvation of protein, lipid and nutrients resulted in the least resistance to virus infection, as expected from honeybee and other systems. Infected bees fed any pollen had lower mortality, but as hypothesized, not all pollen sources are equal in their improvement of response to virus infection. Bees fed *Cistus*, the lowest quality pollen, exhibited significantly higher mortality than polyfloral-fed bees; single-source, *Castanea*-fed bees were intermediate between the two extremes. Together, this shows that even a comparably low-quality pollen improves response to virus, but that a higher quality (*Castanea*) or diverse mixture of pollen is more successful at rescuing bees from virus-induced mortality. Virus titres, however, did not differ across dietary groups. We hypothesize that this is due to nutrition improving bees' tolerance of virus titres, rather than an improved ability to destroy virus particles or simply reduce virus replication; however, more targeted studies are needed to fully test this hypothesis.

Our results also suggest that the availability of trace micronutrients is an important factor in maintaining honeybee nutritional health. To our knowledge, this is the first study to address whether micronutrient deficiencies are associated with honeybee viral pathology. Recent studies suggest micronutrients may be limited in honeybee diets; foraging bees will seek out food containing trace micronutrients including Na and Ca, and shift their preferences over the course of the season [54] and limited micronutrients are associated with reduction or cessation of brood rearing [29,30]. Our data suggest that even when bees receive adequate food quantities, the absence or deficiency of specific micronutrients (here, Ca and Fe) in the diet can weaken bees’ resilience to viral infection. When fed *Cistus* pollen, which we discovered is deficient in both Ca and Fe, bees in cages had reduced survival when challenged with viral infection. However, there was no difference in short-term survival in the absence of viral infection (figure 1), though Di Pasquale *et al*. [24] showed reduced survival between 20 and 50 days. In this experiment, we were able to show that the bees that fed on this pollen also possessed lower levels of Ca and Fe in their bodies (figure 3), suggesting they could have suffered from Ca and/or Fe deficiency. Despite these observations, however, micronutrient levels *per se* were not predictive of virus titre or mortality. This may be because there are also other differences in these pollens; *Cistus* also contains lower protein levels and has different amino acid content [24]. Therefore, our results suggest that any given nutritional component, whether Ca, Fe, or amino acids, may be part of a more complicated profile of constituents. Future work will be necessary to determine the relative value of these nutrients.

Although we have not shown that micronutrient deficiency *per se* is the cause of honeybees' susceptibility to viral infection, studies in humans and other animals suggest micronutrient deficiency can be related to reduced immune function and susceptibility to disease [31–33,55]. In addition, numerous iron-containing proteins are involved in immune function [31], and some insect immune proteins have calcium-dependent activity [56], suggesting a potential underlying mechanism by which micronutrient deficiency could reduce honeybee immune system health. Furthermore, while *Cistus* pollen had no detectable iron content, bees fed this pollen still have intermediate iron content in their bodies, probably present from larval nutrition. Therefore, if larvae are reared in an iron-deficient environment, then emerge into that same environment as adults, this deficiency may be more pronounced. Overall, there has been little work on micronutrient requirements of honeybees [57], but studies on other insects suggest there are strong effects of deficiencies in trace metals and salts on organismal metabolism [32], growth [58] and fecundity [34,58–61]. We suggest further investigations of micronutrient deficiency, along with the possibility of micronutrient supplementation, could be fruitful in improving bee health in the future. Further, future work can focus on better understanding the mechanisms by which these effects might occur.

Our study suggests good nutrition can buffer bees from the negative effects of viruses, but the mechanisms of this effect are still unknown. Under different diets, all virus-inoculated bees’ IAPV titres were elevated compared to uninfected controls, but there were no significant differences in virus titre between the inoculated groups (figure 2). Prevalence of DWV has been shown to actually increase with pollen feeding, possibly due to an increase in the physiological machinery that viruses use to multiply [53]. This suggests that bees fed higher-quality diets may be able to tolerate higher virus titres without exhibiting mortality. While not definitive, we hypothesize that good nutrition allows bees to survive the presence of potentially lethal virus levels. However, future work needs to be done to better understand how honeybees use nutrition to maintain resilience to virus infection.

The same pattern of mortality was not observed in the queenless hives, however, and thus we do not show any evidence of reproducing this phenomenon in the field. There are several reasons that differences in mortality may not have been observed. First, because of the limitations of colony-level analysis (i.e. lower sample size), we were unable to statistically compare mortality between treatment groups. Despite this, we also did not observe any trends in mortality comparable to those found in the cage experiments. This could be because of either colony-level buffering against stress [62], in which larger groups are more resilient than smaller groups or individuals. Furthermore, bees in colonies were infected at seven days of age instead of 24 h, and older bees are probably more resilient against infection as their immune system develops (particularly this inoculum dose, which was optimized for newly emerged bees). Despite the lack of mortality effects, we found that colonies under both extreme nutritional stress (no pollen) and viral infection produced the largest number of bees exiting (and arriving to) the colony (figure 4; electronic supplementary material, figure S3), suggesting increased foraging activity. Previous studies have also shown that virus infection [63] and immune stimulation [64] can affect foraging onset, though these did not incorporate nutritional manipulation. Social insects also have been shown to remove themselves from the social unit altruistically in the face of some stresses [64,65], though this is not completely consistent with our complementary findings on bees arriving.

The data presented here are consistent with the hypothesis that interacting stressors can shift colony division of labour in a way that could lead to the production of more foragers, possibly inducing young bees to forage precociously. These data corroborate a recent model that uses our understanding of honeybee division of labour to explain the mysterious ‘worker disappearance’ syndrome associated with CCD [66]. Perry *et al*. [66] suggest that high levels of stress, including nutritional stress, cause early and elevated foraging by honeybee colonies. If stressors are extreme, this can lead to an imbalanced colony division of labour driven by inefficient precocious foragers, which are less likely to return successfully to the colony. As missing foragers are replaced by additional precocious foragers, extreme circumstances are predicted to lead to rapidly reduced worker populations, and even CCD-like symptoms [66]. Under extreme colony nutritional stress (e.g. extreme pollen dearth), many bees could be induced to forage precociously [48]. If these precocious foragers are also infected with a disease such as IAPV, then they may be impaired and less likely to return from foraging trips. As the colony continually replaces missing foragers, this could potentially lead to rapid depopulation of the worker force in the colony. However, this hypothesis requires the foraging bees to, by some mechanism, disappear, i.e. not return to the hive. In our experiments, this was not observed, as the number of arriving bees was not different between the treatments (electronic supplementary material, figure S2), though this phenomenon should be further investigated in more natural hive settings (i.e. not in an enclosed greenhouse) with larger sample sizes.

## 5. Conclusion

This study presents important new information regarding the effects of multiple, interacting stressors on honeybee health. The main finding of this study is that interactions between stressors can lead to synergistic effects on honeybees' ability to withstand stress; specifically, poor diet quality and virus infection together can lead to extremely elevated levels of bee mortality. In addition to elucidating the effects of interacting stressors on honeybee health, we also provide data suggesting micronutrient deficiencies may be an area of concern for bee health, but more work is needed to understand the context and mode of action of micronutrient effects on bee health. Finally, our data extend beyond cage assays and show that colonies in the field are susceptible to imbalanced division of labour when faced with the dual stresses of poor diet and virus infection. In the context of bee management and conservation, our data suggest good nutrition is an extremely important component of bee health, as high-quality diets appear to buffer bees from other forms of stress [45,67]. Thus, we suggest that a focus on improving access to high-quality diets could act as a ‘safety net’ for honeybees in dealing with other types of stress in their environment, including disease and pesticide exposure [47,68].

## Supplementary Material

Supplementary Methods, Figures S1, S2, S3

## Data accessibility

The datasets generated and/or analysed during the current study are archived through Dryad Digital Repository: http://dx.doi.org/10.5061/dryad.p0t7770 [69].
