# Supplementary material for: Interacting stressors matter: diet quality and virus infection in honeybee health
Source: R Soc Open Sci. 2019 Feb 6;6(2):181803. doi: 10.1098/rsos.181803 (PMC6408407; doi:10.1098/rsos.181803)
Supplement: Supplementary Methods, Figures S1, S2, S3 [file rsos181803supp1.pdf]

Supplementary Materials and Methods:

Virus inoculum production

Honey bees were treated with a virus inoculum identical to that thoroughly described in Carrillo-Tripp et al. [43] In short, sources of IAPV were identified by screening adult workers om field colonies via RT-qPCR (see below); 50 workers from infected colonies were then sampled and homogenized in 100 mL of PBS buffer. Samples were then centrifuged to remove debris and virus particles enriched by 7% polyethylene glycol (PEG) precipitation. Virus particles were then resuspended in TES buffer (10 mM Tris-HCl pH 7.5, 2 mM EDTA, 150 mM NaCl) and concentrated. This starting inoculum was then amplified by injection into white eye honey bee pupae. In short, pupae were carefully excised from comb at the white eye stage, then injected with 1ul of viral concentrate. They were then incubated at 32ºC for 5-7 days, after which they were collected and stored at -70ºC. After several rounds of pupal infections, during with approximately 500 pupae were infected, pupae were homogenized and viral particles enriched as described above. Viral particles were then stored at -70ºC until use. To calculate the percentage of each virus in virion preparations (Acute bee paralysis virus (ABPV), Black queen cell virus (BQCV), DWV, IAPV, Kashmir bee virus (KBV), and sacbrood virus (SBV)), the virus stock was diluted 1:1000 and used directly for RTqPCR (as described below). This inoculum contained 7.7×10^6 genome equivalents (g.e.) (97.94%) SBV, 7.84×10^4 (0.98%) IAPV, 6.16×10^4 (0.77%) DWV, and 2.57×10^4 (0.32%) BQCV. ABPV was below detectable limits. Despite the high presence of other viruses, particularly SBV, this inoculum results in high acute mortality and an increased titer of only IAPV [43].

Virus quantification

Two bees sampled at 36 hpi were processed from 9–10 randomly choses cages (4 or 5 from each replicate). Virus titers were measured via RTq-PCR identically to the methods described in [43] and in the Supplementary Methods. Total RNA was extracted from the bees using TRIzol (Life Technologies), treated with DNAse I and then diluted to 100 ng/µl. Virus quantity is reported as estimated genome equivalents, which was determined via absolute quantification approach by extrapolation to a standard curve of *in vitro* synthesized viral RNA. For each sample, the viruses found in the viral inoculum (BQCV, DWV, IAPV, SBV) were

measured. Melting curves of qPCR products were compared to the viral RNA positive controls for verification.

Micronutrient quantification

From the solid homogenate of bee tissue produced for lipid quantification, another approximately 100 mg subsample was collected and analyzed for concentration of Ca, Cu, Fe, K, Mg, Mn, Na, and Zn as described in [49] and Supplementary Methods. In short, the sample was placed into an acid-washed test tube in concentrated nitric acid (HNO_3) and heated on a block heater, digesting in boiling nitric acid for approximately 30 min. Then, 0.2 ml of a 10 ppm scandium internal standard and 1.3 ml of deionized water was added to each sample. Undigested lipids were separated with HPLC grade chloroform and the aqueous layer measured using ICP-OES (Perkin-Elmer Optima 3000 DV). Quantity was then determined with a calibration curve for each metal adjusted to the scandium internal standard. Concentrations were then calculated by adjusting element content per the original sample mass. This method was also repeated for subsamples from the pollen diets themselves.

Supplementary Figures

Figure S1A

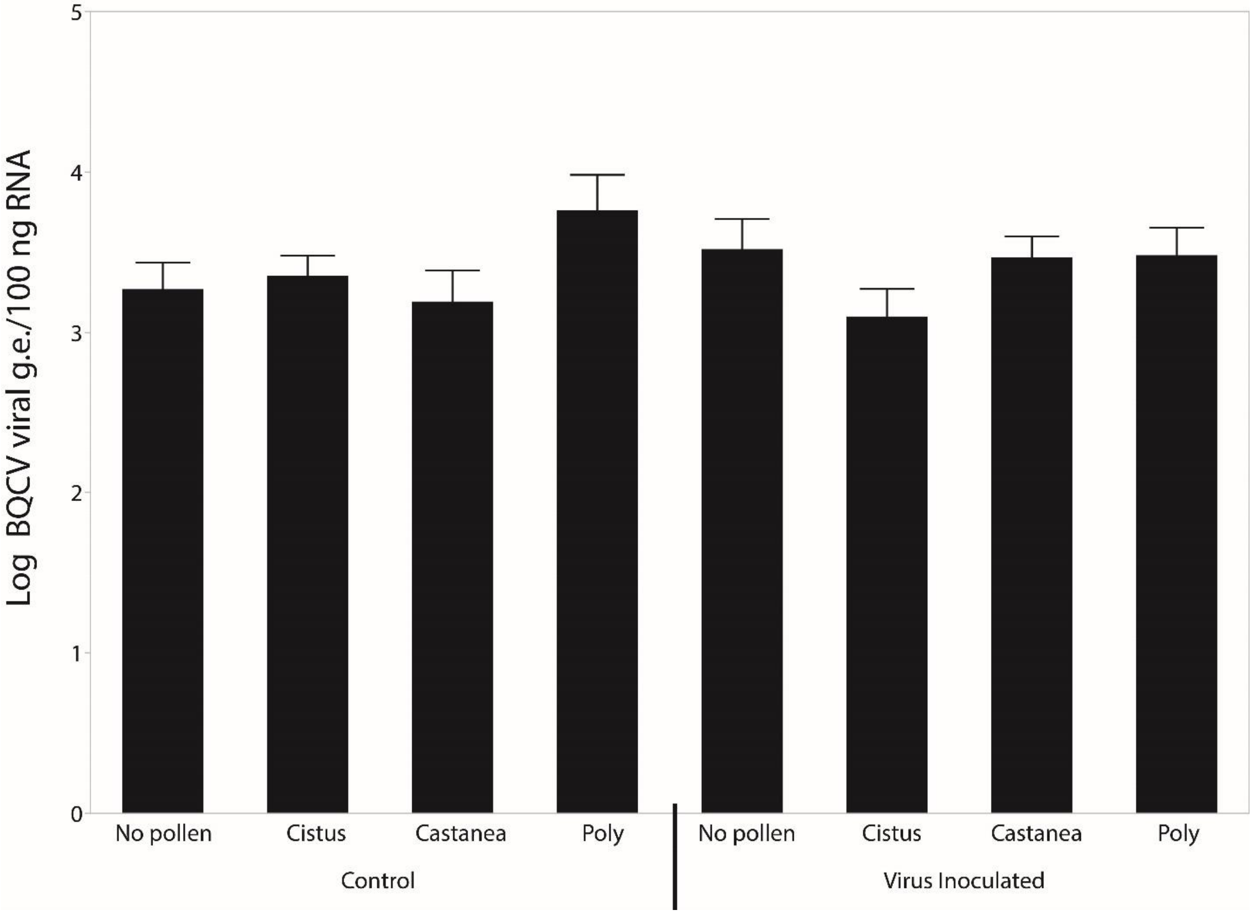

S1B

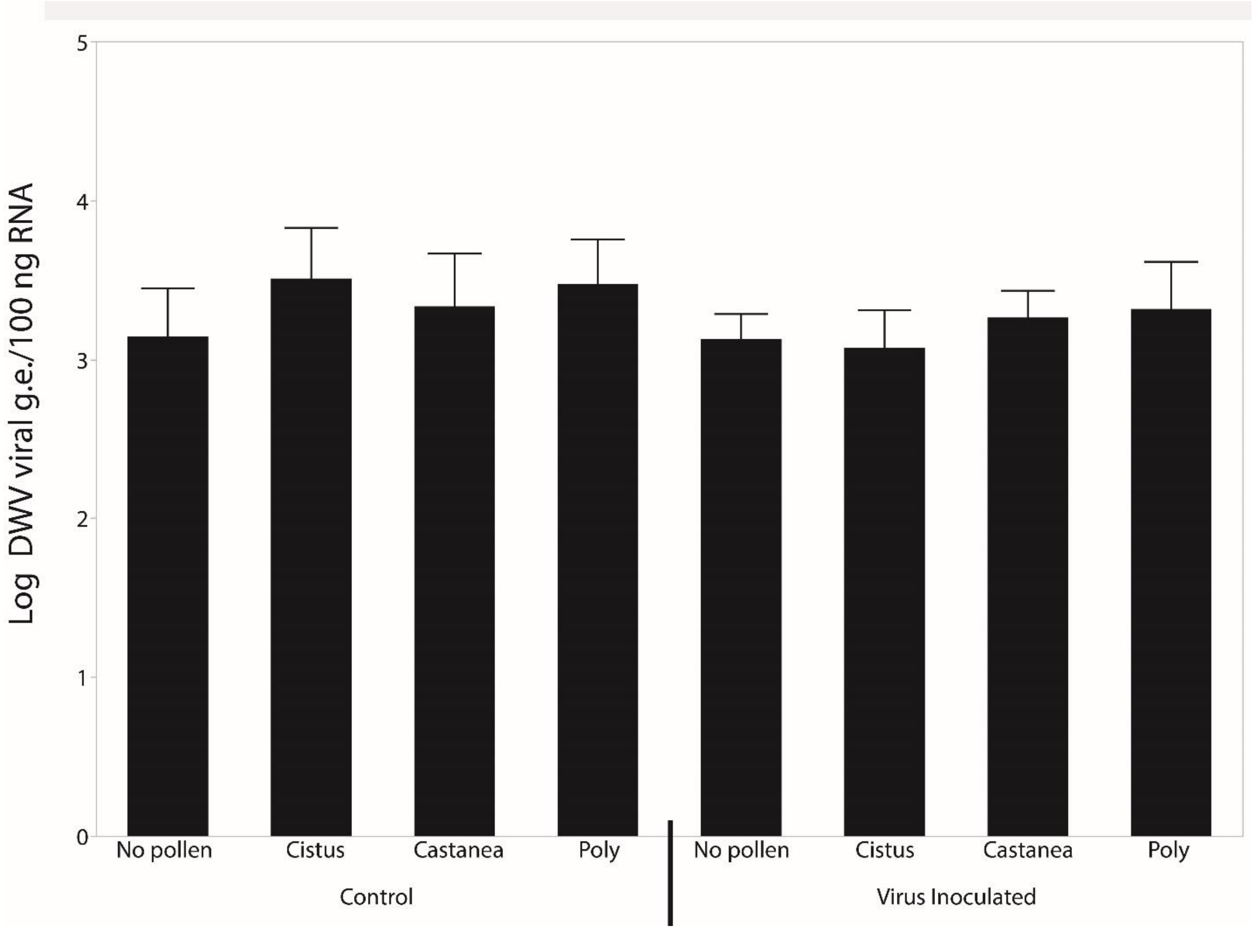

Log DWV viral g.e./100 ng RNA
0
1
2
3
4
5
No pollen
Cistus
Castanea
Poly
No pollen
Cistus
Castanea
Poly
Control
Virus Inoculated

.

S1C

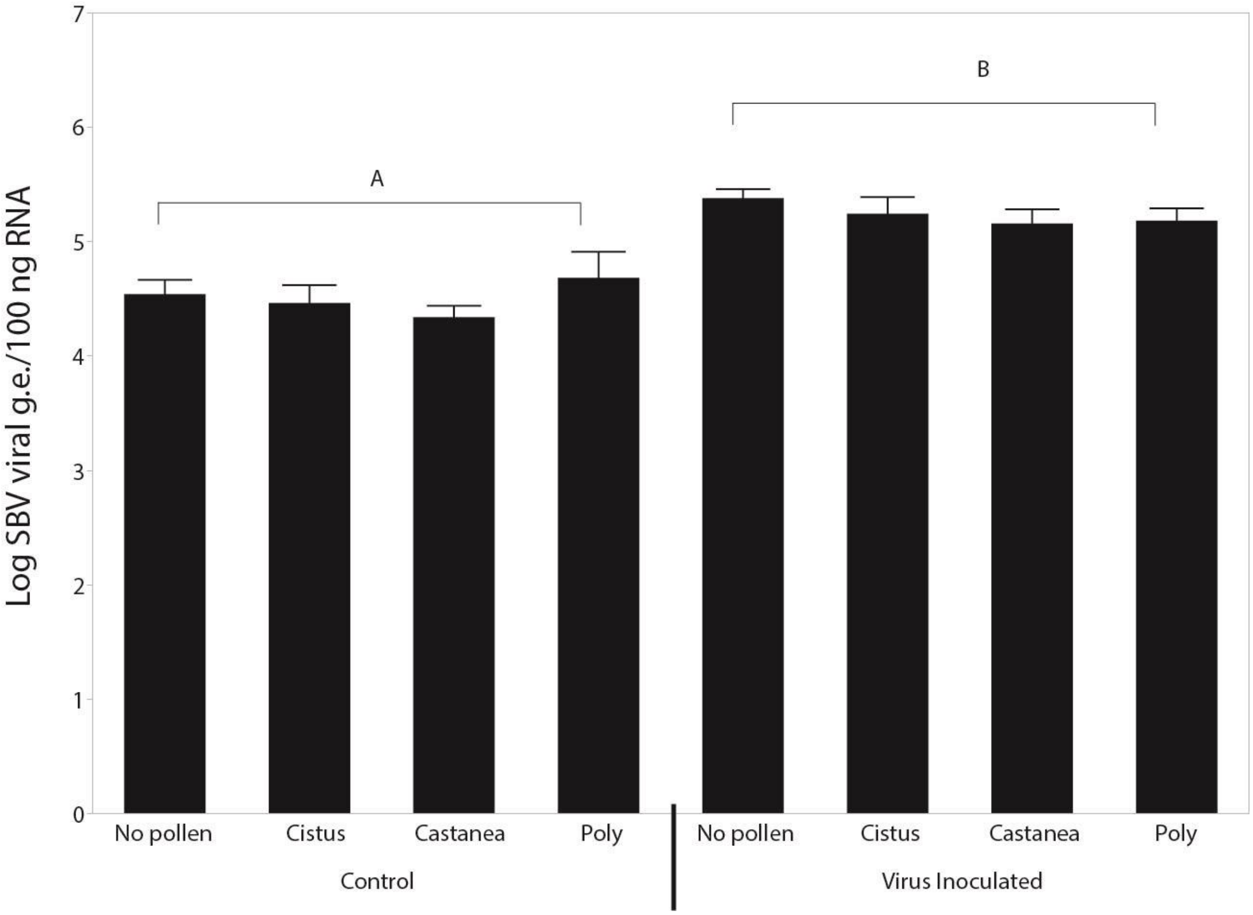

S1 Fig.: Virus load of a)BQCV, b) DWV, and c) SBV expressed as mean +/- S.E. log viral genome equivalents (g.e.) in 100 ng of total RNA at 36 h.p.i from a pool of two live bees sampled per cage. Mean +/- S.E., letters denote significant differences between groups (mixed model ANOVA, Fisher LSD, Benjamini-Hochberg correction, $p<0.05$,).

S2

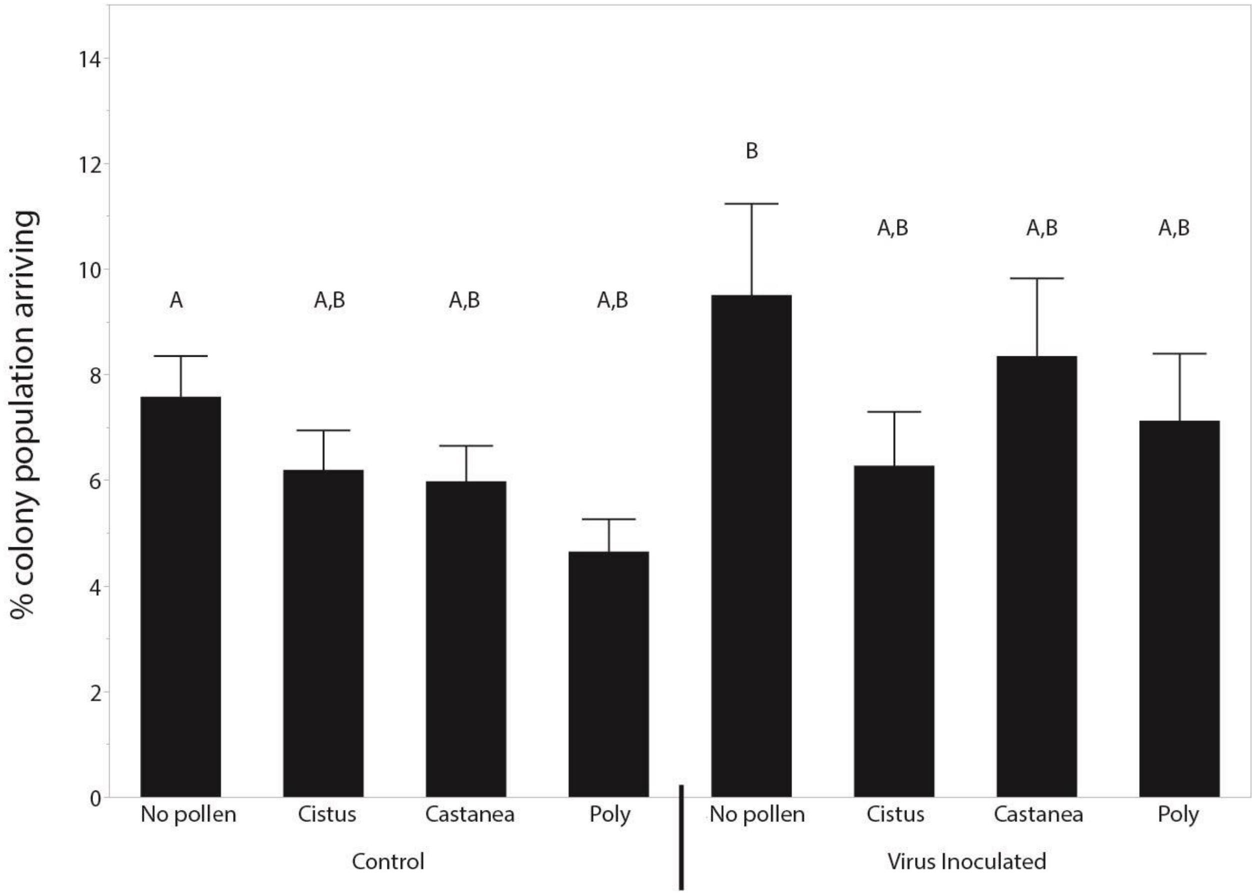

S2 Fig: Percent of a colony's population observed arriving at the hive entrance during 30 minute observation periods repeated over 6 days per colony. Mean +/- S.E., letters denote significant differences between groups (repeated measures ANOVA, Tukey HSD, $p<0.05$).
